# The Combination of MiRNA-196b, LCN2, and TIMP1 is a Potential Set of Circulating Biomarkers for Screening Individuals at Risk for Familial Pancreatic Cancer

**DOI:** 10.3390/jcm7100295

**Published:** 2018-09-20

**Authors:** Detlef K. Bartsch, Norman Gercke, Konstantin Strauch, Ronja Wieboldt, Elvira Matthäi, Vinona Wagner, Susanne Rospleszcz, Agnes Schäfer, Frederike S. Franke, Ioannis Mintziras, Christian Bauer, Tobias Grote, Jens Figiel, Pietro Di Fazio, Andreas Burchert, Silke Reinartz, Elke Pogge von Strandmann, Günter Klöppel, Emily P. Slater

**Affiliations:** 1 Department of Visceral, Thoracic and Vascular Surgery, Philipps University Marburg, Baldingerstrasse, D-35043 Marburg, Germany; bartsch@med.uni-marburg.de (D.K.B.); gercken@staff.uni-marburg.de (N.G.); RonjaWieboldt@web.de (R.W.); Elvira.Matthaei@med.uni-marburg.de (E.M.); vinona.wagner@dkfz.de (V.W.); Agnes.95@web.de (A.S.); Frederike.Franke@uksh.de (F.S.F.); Ioannis.Mintziras@uk-gm.de (I.M.); difazio@med.uni-marburg.de (P.D.F.); 2 Institut für Medizinische Informationsverarbeitung, Biometrie und Epidemiologie, Faculty of Medicine, Ludwig-Maximilians-Universität, Marchioninistr. 15, D-81377 Munich, Germany; strauch@helmholtz-muenchen.de (K.S.); susanne.rospleszcz@helmholtz-muenchen.de (S.R.); 3 Institute of Genetic Epidemiology, Helmholtz Zentrum München–German Research Center for Environmental Health, Ingolstädter Landstr. 1, D-85764 Neuherberg, Germany; 4 Department of Gastroenterology, Endocrinology and Metabolism, Philipps University Marburg, Baldingerstrasse, D-35043 Marburg, Germany; christian.bauer@med.uni-marburg.de (C.B.); tobias.grote@med.uni-marburg.de (T.G.); 5 Department of Diagnostic and Interventional Radiology, Philipps University Marburg, Baldingerstrasse, D-35043 Marburg, Germany; figiel@med.uni-marburg.de; 6 Department of Hematology, Oncology and Immunology, Philipps University Marburg, Baldingerstrasse, D-35043 Marburg, Germany; Andreas.Burchert@med.uni-marburg.de; 7 Center for Tumor and Immune Biology, Philipps University Marburg, Hans-Meerwein-Str. 3, D-35043 Marburg, Germany; silke.reinartz@staff.uni-marburg.de (S.R.); poggevon@staff.uni-marburg.de (E.P.v.S.); 8 Department of Pathology, Technical University Munich, Trogerstr. 18, D-81675 Munich, Germany; guenter.kloeppel@tum.de

**Keywords:** pancreatic ductal adenocarcinoma, LCN2/NGAL, TIMP1, Glypican-1, miRNA-196b, *KRAS* mutation

## Abstract

Individuals at risk (IAR) of familial pancreatic cancer (FPC) are good candidates for screening. Unfortunately, neither reliable imaging modalities nor biomarkers are available to detect high-grade precursor lesions or early cancer. Circulating levels of candidate biomarkers LCN2, TIMP1, Glypican-1, RNU2-1f, and miRNA-196b were analyzed in 218 individuals with sporadic pancreatic ductal adenocarcinoma (PDAC, *n* = 50), FPC (*n* = 20), chronic pancreatitis (*n* = 10), IAR with relevant precursor lesions (*n* = 11) or non-relevant lesions (*n* = 5), 20 controls, and IAR with (*n* = 51) or without (*n* = 51) lesions on pancreatic imaging. In addition, corresponding duodenal juice samples were analyzed for Glypican-1 (*n* = 144) enrichment and *KRAS* mutations (*n* = 123). The panel miR-196b/LCN2/TIMP1 could distinguish high-grade lesions and stage I PDAC from controls with absolute specificity and sensitivity. In contrast, Glypican-1 enrichment in serum exosomes and duodenal juice was not diagnostic. *KRAS* mutations in duodenal juice were detected in 9 of 12 patients with PDAC and only 4 of 9 IAR with relevant precursor lesions. IAR with lesions on imaging had elevated miR-196b/LCN2/TIMP1 levels (*p* = 0.0007) and *KRAS* mutations in duodenal juice (*p* = 0.0004) significantly more often than IAR without imaging lesions. The combination miR-196b/LCN2/TIMP1 might be a promising biomarker set for the detection of high-grade PDAC precursor lesions in IAR of FPC families.

## 1. Introduction

Pancreatic ductal adenocarcinoma (PDAC) is a highly malignant tumor with a poor prognosis. Familial pancreatic cancer (FPC) accounts for 3% to 5% of PDAC cases [1,2]. PDACs are characterized by a progression from pancreatic intraepithelial neoplasia (PanIN) of low-grade over carcinoma in situ (PanIN3) to invasive cancer. The majority of pancreatic specimens of resected FPC individuals reveal multifocal PanINs in addition to small branch-duct intra-ductal papillary mucinous neoplasms (BD-IPMN) [3,4]. In the setting of FPC imaging, BD-IPMNs might be surrogate markers for the presence of high-grade PanIN lesions elsewhere in the gland [5]. Chronic pancreatitis (CP), which is a major differential diagnosis of sporadic PDAC rarely occurs in patients with FPC [6,7]. In contrast, cystic imaging lesions that are potentially BD-IPMNs are visualized in up to 53% of individuals at risk (IAR) from FPC families [7,8].

A recent consensus stated that IAR for the development of PDAC should be screened for a potential surgical treatment [6]. Members of FPC families with at least two affected first-degree relatives are good candidates for screening [6,9]. Only the detection and surgical treatment of T1N0M0 adenocarcinoma and the high-grade precursor lesions PanIN3, main-duct (MD) IPMN, and BD-IPMN with high-grade dysplasia are considered to be a true success of screening [6]. Since these lesions are asymptomatic and very difficult to diagnose with current imaging procedures, there is a need for biomarkers to facilitate screening of IAR in the setting of FPC. At present, there is a paucity of biomarkers that detect early-stage PDAC. These should reliably identify those IAR with PanIN3 or IPMNs with high-grade dysplasia that would allow a potential curative resection. For routine clinical use in FPC screening, biomarkers should consist of a small set that provides quick and reproducible results.

In our previous studies with KPC mice and small patient series LCN2, TIMP1 and miR-196b were identified as potential circulating markers for the early detection of PDAC and its high-grade precursor lesions [10,11]. U2 snRNAs may also serve as novel diagnostic biomarkers for PDAC [12]. Glypican-1 enriched exosomes have been suggested to be reliable biomarkers for the detection of precursor lesions and PDAC [13]. Mutations in *KRAS* were detected in pancreatic juice from 73% of PDAC patients [14]. These potential biomarkers were further evaluated and validated in a total of 218 individuals with PDAC, FPC, CP, IAR with or without pathologically defined pancreatic lesions from FPC families and healthy individuals.

## 2. Materials and Methods

### 2.1. Human Samples

Preoperative blood samples of patients with histologically proven PDAC, FPC, CP, and IAR who underwent pancreatic resection for suspicious lesions were obtained from the prospective tissue bank of the Department of Surgery, Philipps-University Marburg, and the FaPaCa registry. None of the IARs nor the PDAC or CP patients who were resected had any preoperative treatment including neither chemotherapy nor radiotherapy. When available, corresponding early post-operative (7–14 days after resection) samples were also analyzed. Samples from 20 voluntary healthy individuals served as controls. These were not analyzed in our previous pilot analyses [10,11] and constitute a validation set for LCN2, TIMP1, and miR-196b. All tumors were histologically staged, according to UICC-TNM (Union for International Cancer Control; tumor, node, metastasis) classification 2017 [15]. Resection specimens of IAR of FPC families who either underwent total pancreatectomy or partial pancreatic resection were cut into 5 µm sections and screened for the presence of PanINs, IPMNs, and invasive cancer by an experienced pathologist (G.K.). Multifocal PanIN1/2 and intra-ductal papillary mucinous neoplasms (IPMN) with low-grade dysplasia were classified as potentially relevant lesions while only PanIN3 lesions or IPMN with high-grade dysplasia were defined as significant lesions [7]. Serous cystadenomas, multifocal PanIN1 lesions with focal centrolobular fibrosis, and IPMN with low or medium grade dysplasia were considered to be non-relevant lesions. Serous cystadenomas, multifocal PanIN1 lesions with focal centrolobular fibrosis, and IPMN with low or medium grade dysplasia were considered to be non-significant lesions.

In addition, corresponding secretin-stimulated juice samples collected from the duodenum were analyzed for Glypican-1 enriched exosomes (*n* = 144) and *KRAS* mutations (*n* = 123).

Blood and duodenal juice samples were analyzed from 102 IAR who participated in the board-approved, prospective FaPaCa screening program [7,16] with endosonography every 1 to 3 years including secretin-stimulated duodenal juice collection and annual magnetic resonance imaging with magnetic resonance cholangiopancreatography (MRCP). The results were compared to the presence of image-able pancreatic lesions. All subjects gave their informed written consent for inclusion before they participated in the study. The study was conducted in accordance with the Declaration of Helsinki and the protocol was approved by the Ethics Committee of the Philipps University Marburg, Germany (No. 36/1997, amendment 5/2009).

### 2.2. RNA Isolation and Real-Time PCR MiR-196b

The PAXgene^®^ system (Becton Dickinson, Bergen County, NJ, USA) was used to isolate total RNA including miRNA from human samples using the PAXgene^®^ system miRNA kit, according to the manufacturer’s instructions. Real-time PCR was performed in triplicate. miR-196b was amplified after specific reverse transcription using TaqMan MicroRNA assays and a TaqMan^®^ MicroRNA Reverse Transcription Kit (Applied Biosystems, Foster City, CA, USA), according to the manufacturer’s instructions (Applied Biosystems). MiRNAs were normalized to miR-24, which is ubiquitously expressed in normal and pancreatic tissues as previously described [17]. The relative expression was determined by using the delta-delta Ct method and a >35 Ct value indicated negative amplification. A ΔCt value of 6.35 for miR-196b was calculated as a cutoff value that indicates the presence of multifocal PanIN2/3 lesions or PDAC [11]. 

### 2.3. RNU2-1f

The serum levels of RNU2-1f were analyzed in 63 patients using RNA extraction, reverse transcription, and real time PCR. The sera were spiked with 25 fmol of *Caenorhabditis elegans* miRNA-54 (cel-miR-54) that served as a normalization control in real-time PCR, which was previously described [12].

### 2.4. LCN2 and TIMP1

ELISAs of LCN2 and TIMP1 were performed in the preoperative and postoperative sera of enrolled patients, as described previously [10]. Serum samples were diluted and tested for LCN2 and TIMP1 in the corresponding human Quantikine ELISA Kits, DLCN20, and DTM100, respectively (R&D Systems, Wiesbaden, Germany), according to the manufacturer’s instructions. ELISA plates were read on an Emax precision microplate reader (Molecular Devices LLC, Sunnyvale, CA, USA) and the data were analyzed using SoftMAX pro 6.4 software (Molecular Devices LLC). The calculated cutoff values were 102 ng/mL for LCN2 and 273 ng/mL for TIMP1, according to our previously reported analyses [10].

### 2.5. CA 19-9

The serum marker, CA 19-9, was measured by using the electro-chemiluminescence immunoassay (ECLIA), Elecsys^®^, from Roche Diagnostics Ltd. (Rotkreuz, Switzerland), according to the manufacturer’s instructions.

### 2.6. Glypican-1 Circulating Exosomes (crExos) in Serum and Duodenal Juice

Extracellular vesicle (EV) isolation from human serum and duodenal juice samples was performed, as previously described [13]. The samples were filtered through a 0.2 µm pore filter and ultra-centrifuged at 150,000× *g* at 4 °C. This was completed overnight at first and then again for 2 h to obtain the EVs. Alternatively, EVs were isolated by using ExoQuick Solution, as suggested by the manufacturer (System Biosciences, Palo Alto, CA, USA). EVs were attached to 4 mm aldehyde/sulfate latex beads (Invitrogen, Carlsbad, CA, USA) by mixing 30 µg of vesicles with 10 µL volume of beads in 100 µL PBS for 15 min at room temperature with a continuous rotation and then diluted to 1 mL with PBS. The reaction was stopped with 100 mM glycine and 2% BSA in PBS. EV-bound beads were washed, blocked with 10% BSA, and then incubated with anti-GPC1 (R&D Systems, 3 µL of antibody in 20 µL of 2% solution of bovine serum albumin (BSA) for 30 min by rotating at 4 °C. The beads were centrifuged for 1 min at 14,800× *g*, the supernatant was discarded, and the beads were washed in 2% BSA. Alexa-488-tagged secondary antibodies (Abcam, 3 µL of antibody in 20 µL of 2% BSA) were used for 30 min with a rotation at 4 °C. SECONDARY antibody incubation alone was used as a control and to gate the beads with GPC1-bound vesicles. The percentage of positive beads was calculated relative to the total number of beads analyzed per sample (100,000 events). This percentage was referred to as the percentage of beads with GPC1 vesicles. The isolated EVs were characterized by using the ZetaView-Particle-Tracking-Analyzer and found to have the appropriate size for exosomes. In addition, the exosomal proteins CD9 (Merck, Darmstadt, Germany), Flotillin-1 (Abcam, Cambridge, UK), and Glypican-1 (R&D Systems, Wiesbaden, Germany) were detected on Western blots.

### 2.7. KRAS Mutation Analysis of Duodenal Juice

Pancreatic juice secretion was stimulated by infusing synthetic secretin (Secrulux^®^, Sanochemia Diagnostic, Neuss, Germany) and was then collected from the duodenal lumen. Circulating DNA was isolated by using the QIAamp^®^ Circulating Nucleic Acid Kit (Qiagen, Hilden, Germany) and was tested for the presence of mutant *KRAS* by using the *therascreen*^®^ *KRAS* Pyro^®^ Kit (Qiagen), according to the manufacturer’s instructions.

### 2.8. Statistical Analysis

For analyses of miR-196b, LCN2, TIMP1, RNU2-1f, Glypican-1, and CA 19-9 levels in human samples, the Wilcoxon signed rank test as well as the logistic regression modeling was applied. The resulting predicted values were analyzed again by the calculation of a ROC (Receiver Operating Characteristic) curve and the determination of sensitivity, specificity, and AUC (Area Under Curve). The steps were conducted with R version 2.13.1 in addition to PRISM 6 for Mac OS X from GraphPad Software, Inc. (San Diego, CA, USA). A value of *p* < 0.05 was considered significant.

## 3. Results

A summary of the clinical characteristics of 96 patients resected for pancreatic disease and 20 normal controls is presented in Table 1. Subjects with PDAC were significantly older (median 67.5, range 40 to 84 years) than the IAR groups (median 58 and 60 years, *p* < 0.0322). There was no significant difference between groups regarding body-mass-index (BMI), the presence of diabetes, smoking, or a history of pancreatitis.

### 3.1. Serum Analysis of Resected Patients

In the first step, the expression of biomarkers miR-196b, LCN2, TIMP1, RNU2-1f, and CA 19-9 was analyzed in 116 serum samples of patients or IAR who underwent pancreatic resections for PDAC (*n* = 50, 5 stage I, 38 stage II and 7 stage III), FPC (*n* = 20, 6 stage II, 1 stage III and 13 stage IV), CP (*n* = 10), significant lesions (*n* = 5), potentially relevant lesions (*n* = 6), non-relevant lesions such as multifocal PanIN1 or serous cystadenoma (*n* = 5), and healthy controls (*n* = 20), respectively. CA 19-9 was elevated in 85% of patients with PDAC, 100% of FPC patients, 40% of CP patients, but in none of the IAR with or without relevant precursor lesions nor in normal controls. RNU2-1f performed best in discriminating between PDAC and healthy controls as well as between PDAC and CP, but it failed to distinguish between potentially relevant or significant precursor lesions from healthy controls (Table 2 and Table 3). In contrast, the serum levels of Glypican-1 enriched crExos could not distinguish between patients with PDAC or IAR with significant precursor lesions and healthy controls or IAR without significant lesions (Table 2 and Table 3) since 7 of 10 analyzed healthy individuals revealed elevated levels of Glypican-1 crExos.

**Figure 1:**
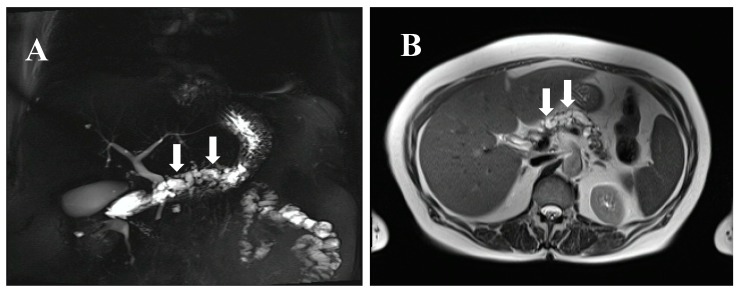
MRCP (magnetic resonance cholangiopancreatography) (**A**) and MRI (magnetic resonance imaging) (**B**) of a 77-year-old female individual at risk (IAR) with a cystic dilated Wirsung duct, a potentially main-duct intra-ductal papillary mucinous neoplasia (IPMN) from a familial pancreatic cancer (FPC) family with four affected relatives. The IAR underwent total pancreatectomy and pathological examination revealed a main duct IPMN with multifocal moderate and severe dysplasia. Preoperative serum levels of miR-196b, LCN2, and TIMP1 were strongly elevated and dropped to normal levels at postoperative day 10. Arrows indicate the cystic dilated Wirsung duct.

The blood levels of miR-196b were elevated in all patients with PDAC, FPC, and in 10 of 11 IAR with potentially relevant or significant precursor lesions compared to normal expression in healthy individuals and IAR with non-relevant precursor lesions (Table 2 and Table 3). Thus, all patients with stage I PDACs (*n* = 5) and all 5 significant lesions and 5 of 6 potentially relevant precursor lesions showed elevation of miR-196b levels. LCN2 and TIMP1 were also present at normal levels in healthy individuals and IAR with non-relevant lesions but were elevated in 84% and 98% of PDAC cases including all 5 stage I PDAC cases and 70% (7 of 10) each of IAR cases with significant and potentially relevant lesions (Table 2). None of these markers alone could discriminate among all groups with high accuracy. However, the marker panel miR-196b, TIMP1, and LCN2 could distinguish significant lesions and stage I PDAC from healthy individuals with an AUC, sensitivity, and specificity of 1 each and a predictive value of 100% (Table 4, Figure 1).

It is of note that postoperative circulating levels of all three markers dropped to normal levels in 22 patients from whom corresponding postoperative serum samples were available (five stage I PDAC, five significant, and five potentially relevant precursor lesions, Table 5). Details of biomarker analyses of IAR who underwent pancreatic resections for suspicious lesions are shown in Table 5.

### 3.2. Duodenal Juice Analysis of Resected Patients

*KRAS* mutations in secretin stimulated duodenal juice were detected in 75% (9/12) of PDAC patients and 44% (4/9) of IAR with either significant (*n* = 2) or potentially relevant (*n* = 2) precursor lesions (Table 2 and Table 5) but not in healthy individuals (0/10). Among these 13 patients, distinct *KRAS* mutations were detected in the following frequencies: G12V, 9 of 13 (69.3%), G12D, 2 of 13 (15.3%), G12S, 1 of 13 (7.6%), and G12C, 1 of 13 (7.6%). Overall, *KRAS* mutations had only a modest sensitivity to detect potentially relevant or significant precursor lesions. As in serum, the levels of Glypican-1 crExos could not discriminate between PDAC, IAR with significant or potentially relevant lesions, and normal controls in duodenal juice samples (Table 2). The analysis of miR-196b, TIMP1, and LCN2 in duodenal juice samples was not possible or gave non-reproducible results.

### 3.3. Biomarker Analysis of IAR with or without Pancreatic Imaging Lesions

IAR who had not undergone surgery included 51 IAR without pancreatic lesions and 51 IAR with pancreatic lesions on imaging. In 47 of 51 (92%) IAR with lesions, at least one cystic lesion was detected measuring between 3 and 14 mm and in 4 (8%) IAR indeterminable lesions of <10 mm were detected. The median follow-up from first imaging to the most recent screening visit was 30 (range of 1 to 64) months. Characteristics and biomarker analyses of IARs are presented in Table 6. There were no differences in gender, BMI, presence of diabetes, or the history of pancreatitis between the two groups. IAR with imaging lesions were significantly older (median 52 vs. 46 years, *p* = 0.006) than IAR without imaging lesions while almost twice as many IAR without imaging lesions had a history of smoking (32% vs. 19%, *p* = 0.157).

According to the cutoff values determined in our previous studies [10,11], miRNA-196b and LCN2 were elevated in 90% (46/51) and 67% (34/51) of IAR with imaging lesions compared to only 20% (10/51) and 33% (17/51) of IAR without imaging lesions (*p* < 0.05), respectively (Table 6). The elevation of these biomarkers was not dependent on age. In contrast, the elevation of TIMP1 levels was not significantly different between the groups (37%, 19/51 vs. 27%, 14/51). Overall, IAR with pancreatic lesions on imaging (*n* = 51) had an elevated marker set miR-196b/LCN2/TIMP1 (19/51, 37%) significantly more often than IAR without imaging lesions (4/51, 8%, *p* = 0.0007). As already stated above, none of the healthy controls revealed elevation of any of the markers. CA 19-9 was elevated in only 3.9% (2/51) of IAR with and in 2% (1/51) of IAR without pancreatic imaging lesions. 

Glypican-1 crExos were enriched in serum from 57% (29/51) of IAR with lesions and 52% (26/50) of IAR without lesions and in 7 of 10 controls tested (Table 6). Levels of Glypican-1 crExos from duodenal juice were also elevated in almost all IAR analyzed as well as in healthy controls (Table 6). 

In addition, 53% (27/51) of IAR with imaging lesions revealed *KRAS* mutations in the duodenal juice compared to only 17% (9/51) of IAR without imaging lesions (*p* = 0.0004) and none of the healthy controls. The most frequently detected *KRAS* mutation was G12V (*n* = 32, 89%), which was followed by G12C (*n* = 2, 5.5%) and G12S (*n* = 2, 5.5%). The presence of a *KRAS* mutation was not dependent on age in either IAR group. Eight of 51 (16%) of IAR with imaging lesions revealed *KRAS* mutations and elevation of the three markers miRNA-196b, TIMP1, and LCN2 compared to 0 of 51 (0%) of IAR without imaging lesions (Table 6).

## 4. Discussion

There has been a growing effort to study circulating biomarkers in PDAC with the aim of identifying noninvasive, reproducible, and cost-effective diagnostic biomarkers that can aid in early diagnosis of PDAC. Biomarkers that reliably indicate the presence of PanIN and IPMN lesions with high-grade dysplasia or early PDAC (T1) allow curative resection, which would be of great value for screening IAR from FPC families. This is especially true in light of a recent review that shows that screening of IAR from FPC families resulted in preventive or curative surgery in only 2.5% (25 of 988) of patients if one considers the resection of histologically confirmed high-grade precursors and stage I PDAC [18]. 

The only biomarker available for PDAC to date is CA 19-9. However, its sensitivity for PDAC and, more importantly, for potentially relevant or significant (e.g., PanIN2 and PanIN 3, respectively) precursor lesions is poor. Therefore, its use as a diagnostic biomarker is not recommended [19]. In the present study, only three of five stage I PDAC and 0 of 11 IAR with potentially relevant or significant precursor lesions had elevated CA 19-9 levels.

Several potential biomarkers for the diagnosis of PDAC have been reported, but rarely for its precursor lesions especially in the setting of FPC [4,13,14,20,21]. Thus, the present study focused on biomarkers that might enable the detection of PanINs and IPMNs with advanced dysplasia as well as early PDAC (stage I) in the setting of FPC. The biomarker set miR-196b/LCN2/TIMP1 distinguished IAR with significant precursor lesions and stage I PDAC from healthy controls with absolute specificity and sensitivity. However, this biomarker set had a limited sensitivity and specificity of 80% each for the discrimination between CP and multifocal PanIN2/3. This reduced sensitivity is negligible in the setting of FPC because individuals with FPC rarely have chronic inflammation of the gland [6,7]. The good performance of the combination of miR-196b, LCN2, and TIMP1 as a potential biomarker panel for the detection of early disease in IAR of FPC families is not surprising. TIMP1 has been proposed to be a potential diagnostic biomarker for PDAC [10,22,23,24,25]. The Alliance of Pancreatic Cancer Consortia for Biomarkers for Early Detection recently stated that, while no biomarker is ready for a validation trial, TIMP1 had sufficiently high sensitivity and specificity to warrant additional research especially in combination with other biomarkers to form a panel [21]. The biomarker panel TIMP1/leucine-rich alpha 2-glycoprotein 1/ CA 19-9 significantly improves the detection of early-stage PDAC [24]. However, patients with precursor lesions were not analyzed in that study. TIMP1 is also part of the CancerSEEK [25].

MiR-196b has previously been shown to be the most selectively and differentially expressed miRNA in micro-dissected PanIN3 lesions [26] and it was the most up-regulated miRNA in 248 PDAC tissues including stage I when compared to normal pancreatic duct cells on an miRNA array analysis [27]. Furthermore, the two-miRNA index “miR-196b-miR-217” was suggested to be a useful tool for distinguishing patients with PDAC from those with a normal pancreas and CP based on a validation in 241 paraffin-embedded pancreato-bilary cancers and 74 benign pancreas tissues [28]. In our former pilot study on transgenic KPC animals and a small patient series, we showed that the combination of miRNAs 196a and 196b reached a sensitivity of 1 and specificity of 0.9 (area under the curve = 0.99) to diagnose PDAC or its high-grade precursor lesions [11].

Transcripts of LCN2 were significantly higher in the majority of solid tumors including PDAC when compared to normal tissues [29]. A recent review stated that the discriminative power of LCN2 between PDAC patients and controls was acceptable, but the diagnostic accuracy remained uncertain [30]. LCN2 discriminated between PDAC and CP [24,29,31] with limited accuracy and was eliminated from validation tests by some groups [24,30]. However, in the setting of FPC, CP plays only a minor role as a differential diagnosis. More importantly, LCN2 expression was detected in the PanIN-stage [31] and it could differentiate between mucinous pancreatic cysts and non-mucinous cysts [32], which suggests that it could be a marker of premalignant changes in the pancreas. Given that TIMP1 and LCN2 may be elevated in other cancer types [29], they are best suited as part of a panel for subjects at increased risk such as those with a history of FPC.

A recent study on 190 patients with PDAC indicated a strong correlation between Glypican-1 crExos and PDAC [13]. We cannot confirm this observation. Glypican-1 crExos were not diagnostic for PDAC or its relevant precursor lesions in the present cohort. Our results are supported by Lai et al. [33] who demonstrated that Glypican-1 crExos were not significantly different between normal controls and pre-resection PDAC samples.

The present analysis is clearly limited by the small number of stage I PDAC and high grade precursor serum samples as in all previous series and, thus, cannot define any biomarker panel for the screening of IAR from FPC families with enough statistical power. However, these samples are extremely rare. According to a recent review on resection results of published, board-approved FPC screening programs, only 2.5% (25 of 988) of IAR revealed high-grade precursor lesions (*n* = 23) or stage I PDAC (*n* = 2) [18]. In the future, these rare samples from board-approved programs should be combined for the evaluation and validation of biomarkers in the setting of FPC screening.

With the present work, only three studies describe molecular analyses on blood and duodenal juice samples of IAR from FPC families undergoing regular screening [14,34]. We demonstrate in this paper that the biomarker panel miR-196b/LCN2/TIMP1 was elevated in IAR with pancreatic lesions significantly more often than in IAR without imaging lesions and normal controls (Table 6). In addition, IAR with imaging lesions revealed *KRAS* mutations in secretin-stimulated duodenal juice more often than IAR without imaging lesions (*p* = 0.0004). This partially supports the results of a previous study on secretin-stimulated duodenal juice samples of 194 IAR from FPC families [14]. These authors also detected *KRAS* mutations significantly more often in IAR undergoing cancer screening than in controls. Next generation sequencing was performed on pancreatic juice samples from IAR undergoing surveillance and patients with PDAC [34]. Mutation concentrations could distinguish patients with PDAC or high-grade dysplasia from other subjects. These studies provide important information but are only descriptive. The only way to prove the diagnostic accuracy of any marker panel such as miR-196b/LCN2/TIMP1 would be to resect the pancreas with a subsequent pathological examination. However, this is not yet ethically justified. Further annual long-term follow-up will determine whether IAR with elevated levels of miR-196b/LCN2/TIMP1 with or without the presence of *KRAS* mutations in duodenal juice will develop significant precursor lesions or PDAC. This is vital since it has been estimated that 15 to 20 years may lapse before early PanIN or IPMN lesions might be indicated by our biomarker set to become PDAC [35,36].

## Figures and Tables

**Table 1:** Patients’ characteristics.

Parameter	Sporadic PDAC	FPC	CP	Healthy Controls	IAR Relevant Lesions #	IAR Non-Relevant Lesions
*n*	50	20	10	20	11	5
Gender, male/female	27/23	7/13	5/5	11/9	2/9	2/3
Age, years, median (range)	67.5 (40 to 84)	63.5 (47 to 82)	52.5 (44 to 75)	47 (21 to 71)	58 (47 to 77)	60 (42 to 61)
BMI, mean ± SD	26.8 ± 4.3	22.2 ± 3.2	24.1 ± 4.6	23.6 ± 1.9	25.1 ± 2.9	27.4 ± 5.1
Smoking history	20/50 (40%)	6/20 (30%)	2/10 (20%)	5/20 (25%)	3/11 (27%)	0/5 (0%)
Pancreatitis	6/50 (12%)	0/20 (0%)	10/10 (100%)	0/20 (0%)	1/11 (9%)	0/5 (0%)
Diabetes	12/50 (24%)	4/20 (20%)	5/10 (50%)	1/20 (5%)	1/11 (9%)	0/5 (0%)

#, relevant lesions include multifocal PanIN2/3 lesions and IPMN with high grade dysplasia, non-relevant lesions include serous cystadenoma and multifocal PanIN 1 lesions with focal fibrosis, PDAC, pancreatic ductal adenocarcinoma, FPC, familial pancreatic cancer, CP, chronic pancreatitis, IAR, individual at risk, BMI, body-mass-index, SD, standard deviation.

**Table 2:** Biomarker results of patients with pathologically defined lesions.

Elevated Biomarker	PDAC * (*n* = 50)	FPC (*n* = 20)	CP (*n* = 10)	PDAC ** Stage I (*n* = 5)	IAR-RL # (*n* = 11)	IAR-NRL # (*n* = 5)	Controls (*n* = 20)
miR-196b	50/50	20/20	4/10	5/5	10/11	0/5	0/20
LCN2	42/50	20/20	2/10	5/5	7/10	0/5	0/20
TIMP1	49/50	20/20	3/10	5/5	7/10	0/5	0/20
Glypican-1	25/29	15/20	7/10	2/2	7/9	2/5	7/10
RNU2-1f	15/15	10/10	0/21	n.a.	0/7	n.a.	0/10
CA 19-9	39/46	14/14	4/10	3/5	0/11	0/5	0/3
miR-196b + LCN2 + TIMP1	41/50	20/20	0/10	5/5	7/10	0/5	0/20
*KRAS* in duodenal juice	9/12	n.a.	n.a.	1/1	4/9	n.a.	0/10
Glypican-1 in duodenal juice	9/9	n.a.	n.a.	2/2	6/6	n.a.	5/5

*, PDACs include 5 stage I, 38 stage II, and 7 stage III tumors that were potentially curative resected. FPCs include 1 stage IIA, 5 stage IIB, 1 stage III, and 13 stage IV tumors, **, These 5 stage I PDACs are included in the total of 50 PDACs, #, relevant lesions (RL) include potentially relevant, PDAC, pancreatic ductal adenocarcinoma, IAR-RL, individual at risk-relevant lesions, n.a., not available.

**Table 3:** Performance of serum biomarkers in a combined validation set.

Comparison	Marker	*p*	AUC (95% CI)	Sensitivity at 95% Specificity	Specificity at 95% Sensitivity
PDAC vs. Healthy control	miR-196b	0.0051	0.8938 (0.7395 to 1.048)	80%	50%
	TIMP1	<0.0001	0.9617 (0.9125 to 1.011)	93.6%	70%
	LCN2	0.0007	0.9500 (0.8477 to 1.052)	50%	90%
	RNU2-1f	<0.0001	1.0000 (1.0000 to 1.000)	100%	100%
	Glypican-1	0.2700	0.6106 (0.4332 to 0.7881)	31%	0%
FPC vs. Healthy control	miR-196b	0.0006	0.9716 (0.9052 to 1.038)	90%	70%
	TIMP1	0.0001	0.9600 (0.8871 to 1.033)	86%	50%
	LCN2	<0.0001	0.9786 (0.9289 to 1.028)	78%	90%
	RNU2-1f	0.0002	1.0000 (1.0000 to 1.000)	100%	100%
Relevant lesions vs. Healthy controls	miR-196b	0.0011	0.9722 (0.9034 to 1.041)	88.9%	75%
	TIMP1	0.0048	0.9600 (0.8633 to 1.057)	80%	80%
	LCN2	0.0024	0.9667 (0.8857 to 1.048)	67%	90%
	RNU2-1f	0.8073	0.537 (0.2468 to 0.8247)	14.3%	10%
	Glypican-1	0.9717	0.5046 (0.2438 to 0.7655)	11.1%	16.7%
Significant lesions vs. Healthy controls	miR-196b	0.0066	1.0000 (1.0000 to 1.000)	100%	100%
High grade only	TIMP1	0.0047	1.0000 (1.0000 to 1.000)	100%	100%
	LCN2	0.0047	1.0000 (1.0000 to 1.000)	100%	100%
	RNU2-1f	0.3580	0.6625 (0.3679 to 0.9571)	25%	50%
PDAC vs. chronic pancreatitis	miR-196b	0.0539	0.7550 (0.5074 to 1.003)	60%	70%
	TIMP1	0.0126	0.8300 (0.6132 to 1.047)	10%	80%
	LCN2	0.0015	0.920 (0.7673 to 1.073)	20%	90%
	RNU2-1f	<0.0001	1.0000 (1.0000 to 1.000)	100%	100%
	Glypican-1	0.9615	0.5052 (0.2850 to 0.7213)	10.3%	0%
FPC vs. chronic pancreatitis	miR-196b	0.0411	0.7636 (0.5475 to 0.979)	55%	0%
	TIMP1	0.0078	0.8200 (0.6232 to 1.017)	21%	60%
	LCN2	0.0004	0.9286 (0.8029 to 1.054)	36%	80%
	RNU2-1f	<0.0001	1.0000 (1.0000 to 1.000)	100%	100%

PDAC, pancreatic ductal adenocarcinoma, FPC, familial pancreatic cancer, AUC, area under curve, CI, confidence interval.

**Table 4:** Performance of the serum biomarker panel miR-196b + TIMP1 + LCN2.

Comparison	*p*	AUC (95% CI)	Sensitivity at 95% Specificity	Specificity at 95% Sensitivity
PDAC vs. Healthy control	0.0012	0.93 (0.8162 to 1.044)	80%	80%
FPC vs. Healthy control	0.0004	0.97 (0.9071 to 1.033)	80%	80%
Significant lesions and stage I PDAC vs. healthy controls	0.029	1 (1.000 to 1.000)	100%	100%
PDAC vs. chronic pancreatitis	0.0025	0.9 (0.7559 to 1.044)	50%	80%
FPC vs. chronic pancreatitis	0.0007	0.95 (0.8622 to 1.038)	80%	80%

PDAC, pancreatic ductal adenocarcinoma, FPC, familial pancreatic cancer, AUC, area under curve, CI, confidence interval.

**Table 5:** Detailed biomarker results in IAR with histologically defined pancreatic lesions (*n* = 16) compared to stage I PDAC and controls.

IAR with Histologically Verified Lesions	Pre-OP *KRAS* Mutation in Duodenal Juice	Pre-OP Elevated miR-196b	Pre-OP Elevated LCN2	Pre-OP Elevated TIMP1	Pre-OP Elevated miR-196b + LCN2	Pre-OP Elevated miR-196b + TIMP1	Pre-OP Elevated LCN2 + TIMP1	Pre-OP Elevated miR-196b + LCN2 + TIMP1	Post-OP not Elevated miR-196b, TIMP1, LCN2 *
Sporadic stage I PDAC (*n* = 5)	1/1	5/5	5/5	5/5	5/5	5/5	5/5	5/5	5/5
PanIN3 (*n* = 3)	1/3	3/3	3/3	3/3	3/3	3/3	3/3	3/3	3/3
IPMN with HGD (*n* = 2)	1/1	2/2	2/2	2/2	2/2	2/2	2/2	2/2	2/2
Multifocal PanIN2 or IPMN with MGD (*n* = 6)	2/5	5/6	2/5	2/5	2/5	2/5	2/5	2/5	5/5
PanIN 1 or focal fibrosis (*n* = 2)	n.a.	0/2	0/2	0/2	0/2	0/2	0/2	0/2	n.a.
Serous cystadenoma (*n* = 3)	n.a.	0/3	0/3	0/3	0/3	0/3	0/3	0/3	n.a.
Normal controls (*n* = 20)	0/10	0/20	0/20	0/20	0/20	0/20	0/20	0/20	n.a.

PanIN, pancreatic intraepithelial neoplasia, IPMN, intraductal papillary mucinous neoplasia, MGD, moderate grade dysplasia, HGD, high grade dysplasia, n.a., not available, *, only potentially curative resected patients with pre-operative and postoperative serum samples, IAR, individual at risk, PDAC, pancreatic ductal adenocarcinoma, OP, operation.

**Table 6:** Characteristics and biomarker analysis of IAR with or without imaging lesions who did not undergo surgery.

Parameter	IAR with Imaging Lesions (*n* = 51)	IAR without Imaging Lesions (*n* = 51)	Controls (*n* = 20)	*p*-Value IAR with Lesions vs. IAR without Lesions
Gender (male/female)	23/28	22/29	11/9	*p* = 1.0
Age, years, median (range)	52 (30 to 74)	46 (27 to 63)	47 (21 to 71)	*p* = 0.0006
BMI, mean ± SD	25.8 ± 3.6	25.0 ± 4.3	23.6 ± 1.9	*p* = 1.0
Smoking history	19% (9/47)	32% (14/43)	25% (5/20)	*p* = 0.157
Diabetes	4.2% (2/48)	4.3% (2/46)	5% (1/20)	*p* = 1.0
History of pancreatitis	4.2% (2/48)	4.3% (2/46)	0% (0/20)	*p* = 1.0
miR-196b elevated *	46/51 (90%)	10/51 (20%)	0/20	*p* < 0.0001
LCN2 elevated *	34/51 (67%)	17/51 (33%)	0/20	*p* = 0.0014
TIMP1 elevated *	19/51 (37%)	14/51 (27%)	0/20	*p* = 0.397
crExosGlypican-1 enriched *	29/51 (57%)	26/50 (52%)	7/10 (70%)	*p* = 0.691
CA 19-9 elevated *	2/51 (3.9%)	1/51 (2%)	0/3	NS
miR-196b + LCN2 + TIMP1 elevated *	19/51 (37%)	4/51 (8%)	0/20	*p* = 0.0007
*KRAS* mutated in duodenal juice	27/51 (53%)	9/51 (17%)	0/20	*p* = 0.0004
crExos Glypican-1 enriched in duodenal juice	20/20 (100%)	15/17 (88%)	3/3	*p* = 0.204
*KRAS* mutated in duodenal juice + miR196b/LCN2/TIMP1 elevated	8/51 (16%)	0/51 (0%)	0/20	*p* = 0.0058

*, as determined in blood, bold, statistically significant, mut., mutation, IAR, individual at risk, BMI, body-mass-index, NS, not significant.
